# Complexation of Polypeptides and Polypeptoids with Nucleic Acids: Does Chirality Matter for Salt Stability and Morphology?

**DOI:** 10.3390/biom16091315

**Published:** 2026-09-10

**Authors:** Kimiasadat Mirlohi, Anuja Thapa, Donghui Zhang, Whitney C. Blocher McTigue

**Affiliations:** 1 Department of Chemical and Biomolecular Engineering, Lehigh University, Bethlehem, PA 18015, USA; 2 Department of Chemistry and Macromolecular Studies Group, Louisiana State University, Baton Rouge, LA 70803, USAdhzhang@lsu.edu (D.Z.)

**Keywords:** polyelectrolyte complexation, coacervation, nucleic acids, polypeptides, polypeptoids, chirality

## Abstract

Liquid–liquid phase separation (LLPS), especially via coacervation, offers a novel drug delivery strategy by encapsulating therapeutic agents within phase-separated droplets, thereby improving stability, solubility, and controlled release. Polypeptides and polypeptoids are ideal biomaterials for these systems due to their versatility and tunable properties. Polypeptoids are particularly advantageous, offering enhanced enzymatic resistance and greater control over molecular interactions, making them suitable for complexation studies. This research explores the binary complexation of L-, D,L-, and N-substituted (peptoid) poly-lysine with nucleic acids, specifically two lengths of salmon sperm dsDNA and baker’s yeast tRNA, and two nucleotides, adenosine triphosphate (ATP) and cytidine triphosphate (CTP). By adjusting the charge fractions, we studied the morphology of the complexes and tested their salt resistance under different ionic conditions. Results show that dsDNA forms precipitates with lysine polypeptides and polypeptoids, whereas tRNA forms coacervate droplets, likely due to differences in secondary structure. Both nucleotides formed coacervates in all systems. L-homochiral poly-lysine complexes are the most salt-stable, followed by racemic poly-lysine, with N-substituted polymers being the least stable as ionic strength rises. For all dsDNA and tRNA systems, the complexes remained under physiologically relevant salt concentrations. These results highlight the role of salt and polymer structures in modulating complexation. The study offers insights into nucleic acid complexation, with implications for nucleic acid encapsulation and stabilization.

## 1. Introduction

In cells, liquid–liquid phase separation (LLPS) can organize proteins and RNA into dynamic, membrane-free compartments, as shown by the liquid-like behavior and controlled condensation and dissolution of P granules [1]. Researchers have increasingly explored these same physical principles for drug delivery applications [2]. A subset of LLPS, known as coacervation, arises from electrostatic interactions between polyelectrolytes, leading to phase-separated droplets that can encapsulate therapeutic compounds [3]. This phenomenon offers a compelling strategy for improving the solubility, stability, and controlled release of both small molecules and biologics [4]. The ability of coacervates to spatially organize biomolecules via weak, non-covalent interactions plays a crucial role in protecting and efficiently delivering therapeutics, including peptides, proteins, and nucleic acids [5]. Recent research has underscored the potential of coacervation to enhance the stability of nucleic acid-based therapeutics, such as dsDNA and tRNA, by shielding them from environmental degradation [6]. Through the formation of protective biomolecular condensates, coacervation can mitigate enzymatic breakdown and improve both the thermal stability and bioavailability of nucleic acids [7]. These findings highlight the expanding role of coacervation in therapeutic formulations, particularly in the development of gene therapies and nucleic acid-based vaccines, where maintaining biomolecule integrity is critical for efficacy.

The effectiveness of coacervation in drug delivery is largely dictated by the structural and chemical properties of the polymers involved [8]. Factors such as polymer length, charge distribution, and hydrophobicity significantly influence the extent of phase separation and the morphology of the resulting condensates [9,10]. Consistent with this broader principle, studies of protein condensates have shown that both interaction valence and the linear patterning of cohesive residues govern phase separation, illustrating how macromolecular architecture can encode condensate behavior [11]. Another key consideration is chirality, the property of a molecule that is not superimposable on its mirror image. Chirality introduces an additional layer of complexity to coacervate behavior, as homochiral, mixed-chiral, and achiral polymers exhibit distinct interaction patterns that can impact stability, binding affinity, and functional performance in biological systems [12,13]. These differences directly affect the safety and efficacy of therapeutic formulations, making chirality a critical design parameter in drug development.

Beyond polymer characteristics, external factors such as ionic strength, temperature, and pH also govern phase separation behavior [14]. The presence of salt, a ubiquitous component of biological environments, can significantly alter LLPS dynamics by modulating electrostatic interactions [15]. Variations in salt concentration influence phase stability through charge screening and competitive binding effects, potentially leading to morphological transitions or even phase dissolution [16]. As such, understanding the salt resistance limits of coacervate systems is essential for engineering phase-separated complexes that retain their functionality across diverse ionic conditions. By systematically examining how salt influences phase behavior, researchers can fine-tune coacervation processes to develop robust, salt-tolerant drug delivery platforms.

Polypeptides and polypeptoids (structurally related polymers that lack backbone chiral centers due to N-substitution) have gained attention as promising candidates for studying complex coacervation [17,18]. Their diverse chemical architectures and tunable physicochemical properties make them valuable tools for probing phase separation principles and biomolecular interactions [19]. The ability to precisely control attributes such as chain length, charge density, and hydrophobicity enables the design of tailored coacervate systems for biomedical applications. Notably, polypeptoids offer distinct advantages over traditional polypeptides. N-substitution of peptoids provides enhanced resistance to enzymatic degradation and enables finer control over steric and electrostatic properties [19]. While limited hydrolytic degradability may restrict in vivo use in certain applications, oxidative degradation pathways remain possible [20], and this stability can be advantageous in applications that require extended material lifetime or structural integrity, such as implant coatings, biosensors, or long-term delivery matrices [21,22]. These features make polypeptoids particularly attractive for drug delivery applications, where stability, solubility, and bioavailability are key considerations.

Additionally, the biocompatibility and biodegradability of polypeptides and polypeptoids make them well-suited for biomedical applications beyond drug delivery, including tissue engineering and biomaterials development [23]. Their structural versatility allows for the creation of responsive delivery systems that adapt to biological stimuli. Importantly, the morphology of coacervate complexes is highly dependent on the chirality of the interacting polymers. While homochiral polymer systems often favor solid precipitation due to backbone alignment, mixed-chiral or achiral compositions tend to promote LLPS, providing insights into both fundamental coacervation principles and potential therapeutic innovations [12,13].

While our previous work compared polypeptide–polypeptide complexes to assess chirality and salt effects [13], the present study extends this approach to polymer–nucleic acid systems, introducing a polyanionic partner whose backbone geometry, base stacking, and secondary/tertiary folding introduce additional variables not present in polymer–polymer systems [7]. This expansion allows us to ask how nucleic acid structure influences salt tolerance and morphology in coacervates with varied backbone chemistries. It also asks whether chiral centers are necessary for the levels and types of complexations observed with nucleic acids and peptides formed from standard amino acids.

In this work, we study binary systems of lysine homopolymers with varying chiral centers complexed with two lengths of salmon sperm dsDNA and baker’s yeast tRNA as model nucleic acids. We chose these two nucleic acids for their distinct structures: a double helix for dsDNA and a cloverleaf conformation for tRNA. Cellular studies also demonstrate that RNA can actively regulate condensate phase behavior rather than functioning only as cargo; changes in RNA concentration and RNA-binding capacity can shift RNA-binding proteins among soluble, liquid-condensed, and solid-like states [24]. Our results highlight the significant influence of secondary structure on complex morphology and phase behavior. We compared these results to nucleotide (ATP and CTP) systems to further investigate whether single- or double-ringed monomers affect complexation more specifically. To probe how these systems respond to increasing ionic strength, we conducted salt resistance studies using NaCl. These experiments reveal that chiral centers and backbone alignment play central roles in determining the stability of polymer–nucleic acid complexes against salt. By identifying the fundamental characteristics that govern complexation profiles, our findings offer guidelines for the rational formulation of nucleic acid-based therapeutics and can inform material selection and design strategies to achieve more stable and efficient treatments. Specifically, we view our results for bulk coacervates as a starting point for systems employing block copolymers and other architectures that yield coacervate micelles for drug delivery. Our focus on the complexation of our lysine polymers with model dsDNA, tRNA, and nucleotides elucidates how factors such as chirality, the presence of a double helix or cloverleaf structure, and the ring structure of the base nucleotides alter the phase space.

## 2. Materials and Methods

### 2.1. Materials

Poly(L-lysine bromide) (PLK_50_) (*M*_n_ = 13.3 kg/mol, Ð = 1.04), poly(D,L-lysine trifluoroacetate) (PRK_50_) (*M*_n_ = 11.0 kg/mol, Ð = 1.09), and poly(*N*-4-ammonium-butyl glycine trifluoroacetate) (PNK_50_) (*M*_n_ = 12.6 kg/mol, Ð = 1.01) with a number average degree of polymerization (DP_n_) of 50 were synthesized by benzylamine-initiated ring-opening polymerization of the corresponding amino-acid-derived *N*-carboxyanhydride monomers, followed by deprotection to expose the ionizable side chains. The polymers were purified by precipitation, redissolution, and lyophilization, and their composition and molecular characteristics were confirmed by ^1^H NMR and SEC-DRI-MALS. RP-HPLC analysis showed comparable elution times for all three polymers, suggesting no significant difference in their hydrophobicity (Figure S1). Full synthetic procedures and characterization details have been reported previously [13]. Salmon sperm double-stranded DNA with ≤2000 bp (dsDNA-2000) and 200–500 bp (dsDNA-350) and baker’s yeast transfer RNA (tRNA) with 75–80 bp were obtained from Thermo Fisher Scientific, Waltham, MA, USA, and diluted to 10 mM with autoclaved deionized water. Adenosine 5’-(tetrahydrogen triphosphate) (ATP) and cytidine 5’-(tetrahydrogen triphosphate) (CTP) were purchased from Thermo Fisher Scientific as 100 mM stocks and diluted to 10 mM with autoclaved deionized water. Stock polymer solutions (10 mM, calculated on a monomer basis) were prepared gravimetrically in deionized, ultrapure water (Aries FilterWorks, Camden, NJ, USA, 18.2 MW-cm) and adjusted to the target pH (±0.03 units) using small additions of 1 M or 0.1 M HCl and NaOH (Mettler Toledo, Columbus, OH, USA, InLab Expert Pro-ISM). 2-[4-(2-hydroxyethyl)piperazin-1-yl]ethanesulfonic acid (HEPES) buffer (0.5 M, pH 7.0) was prepared in deionized water. Sodium chloride (NaCl) was purchased from Thermo Fisher Scientific and dissolved to make 2 M stock solutions. 

### 2.2. Sample Preparation, Turbidity Measurements, and Optical Microscopy

Coacervate samples were prepared at room temperature in 1.5 mL microcentrifuge tubes (Thermo Fisher Scientific) by sequentially adding ultrapure water and HEPES buffer, then adding the negative polymer (dsDNA, tRNA, ATP, or CTP) and vortexing. The poly-lysine variants (PLK_50_, PRK_50_, or PNK_50_) were then added and immediately mixed to ensure rapid, uniform complexation. Unless otherwise noted, experiments were conducted at a total polymer concentration of 2 mM (on a monomer basis) in 10 mM HEPES, with a final volume of 120 μL. We calculated the amount of each polymer based on the desired charge fraction ($f_{+}$) and describe the charge fraction calculation in the SI (SI Charge Fraction Calculations, SI Equation (S3)).

For stoichiometric studies, 15 mixtures were prepared across a range of polymer-to-nucleic acid nominal charge ratios (0.1 to 0.9), and the charge fraction at which turbidity was maximized was observed. Salt resistance experiments were performed using mixtures fixed at a charge fraction of 0.5. Thirty samples were prepared for each series of salt studies, increasing NaCl concentration from 0 to 1500 mM, with one blank sample including only HEPES buffer at 10 mM in water. In the salt experiments, salt was added between buffer and polyanion. All experiments were performed in triplicate.

For turbidimetric analysis, three 35 μL aliquots of each sample were transferred into a 384-well plate (Falcon, Corning Life Sciences, Tewksbury, MA, USA). Absorbance was recorded at 562 nm using a BioTek Cytation 5 microplate reader (Agilent Technologies, Santa Clara, CA, USA), and turbidity was calculated as −ln(I/I_0_), where I_0_ represents the incident light intensity and I the transmitted intensity. Measurements were baseline-corrected against aqueous samples with only 10 mM buffer as controls. Following turbidimetry, samples were examined by optical microscopy (Echo Rebel, Discover Echo Inc., San Diego, CA, USA) to verify complexation and assess droplet morphology.

### 2.3. Statistical Analysis

All data are presented as mean values with error bars indicating the standard deviation. Each condition was evaluated using three independent biological replicates, each with three technical replicates and three plate reads per sample, yielding an effective sample size of N = 27 per sample.

## 3. Results

### 3.1. Complexation Extent and Morphology of Nucleic Acid Complexes

**Figure 1:**
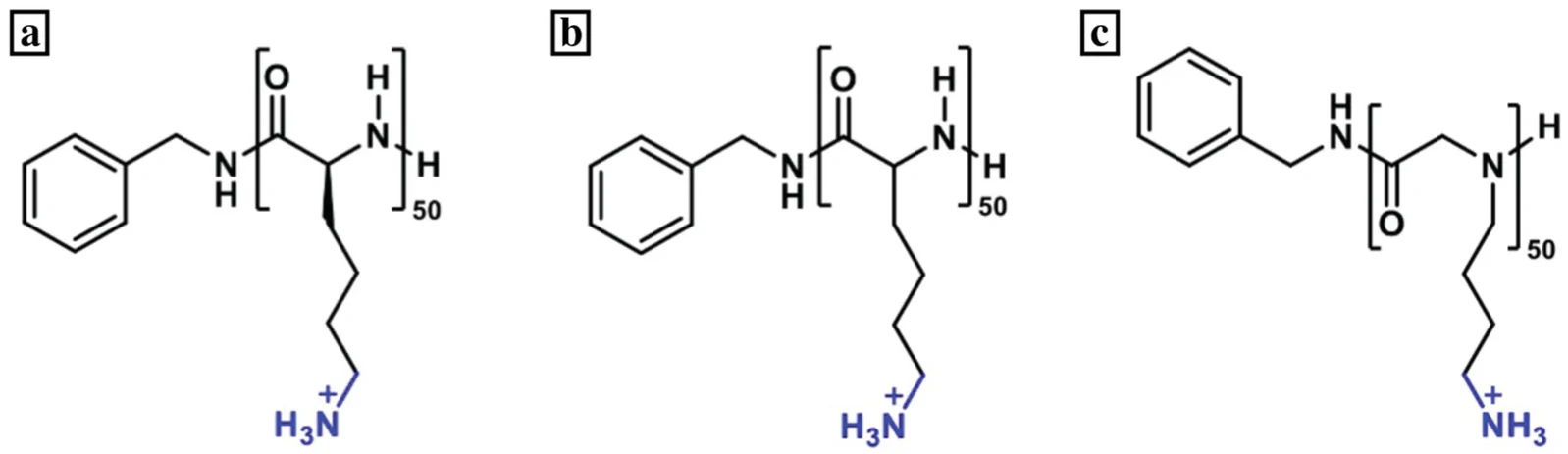
Chemical structures of cationic polypeptide and polypeptoid polymers synthesized by the ring-opening polymerization method of (**a**) poly(L-lysine bromide) (PLK); (**b**) poly(D,L-lysine trifluoroacetate) (PRK); and (**c**) poly(N-4-aminobutyl glycine trifluoroacetate) (PNK).

**Figure 2:**
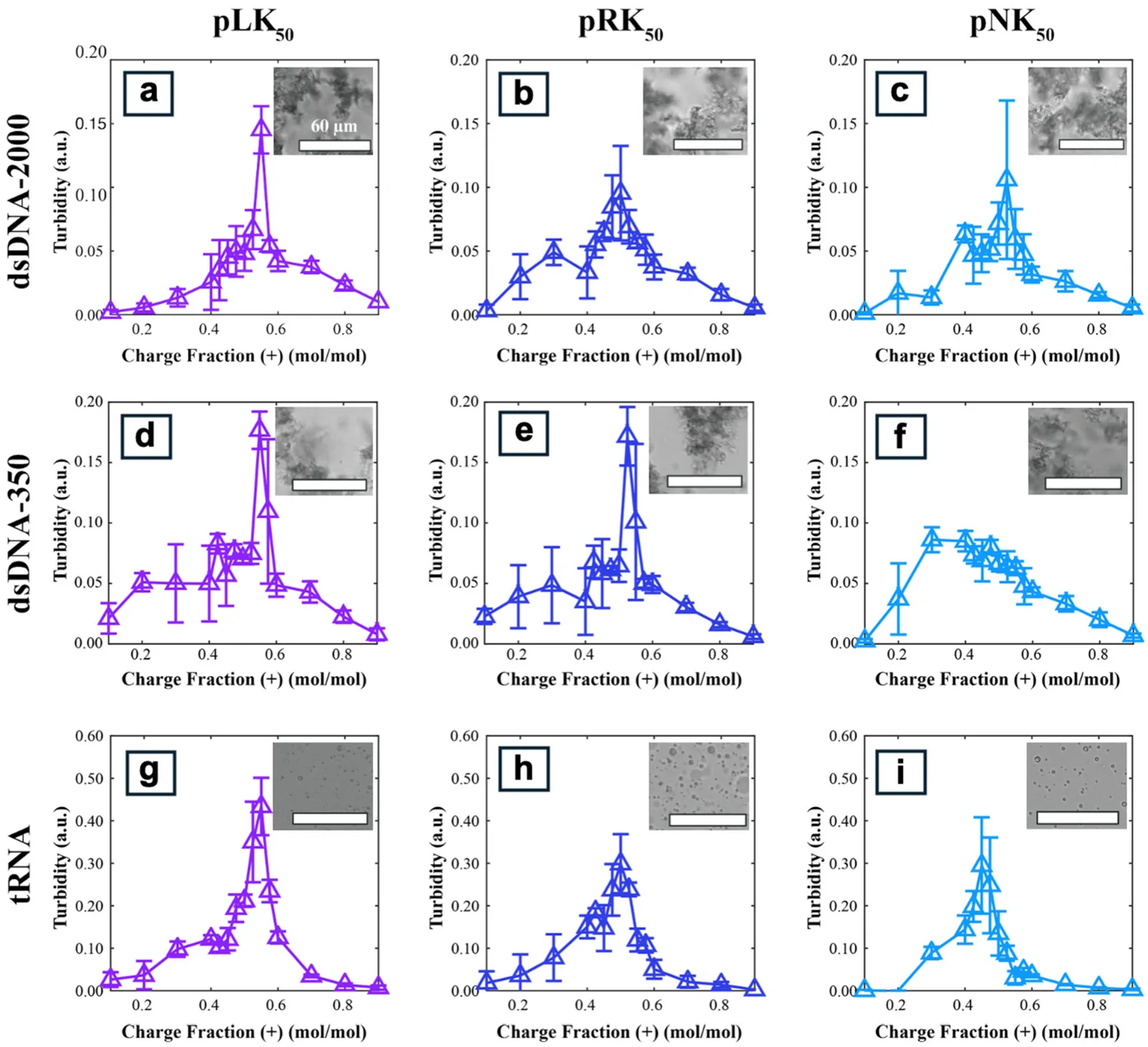
Turbidity measurements and micrographs of three poly-lysine variants (pLK_50_ in purple, pRK_50_ in dark blue, pNK_50_ in light blue) and various nucleic acid combinations at varying polycation charge fractions. Turbidity versus charge fraction (+) (mol/mol) curves show the changes in turbidity readings and, consequently, the complexation. Micrographs were taken at each system’s respective peak turbidity. (**a**) dsDNA-2000+PLK_50_, (**b**) dsDNA-2000+PRK_50_, (**c**) dsDNA-2000+PNK_50_, (**d**) dsDNA-350+PLK_50_, (**e**) dsDNA-350+PRK_50_, (**f**) dsDNA-350+PNK_50_, (**g**) tRNA+PLK_50_, (**h**) tRNA+PRK_50_, and (**i**) tRNA+PNK_50_. Scale bars represent 60 µm in all micrographs. Note that dsDNA 200–500 dp is denoted as dsDNA-350, and dsDNA with dp ≤ 2000 is denoted as dsDNA-2000.

To investigate how polymer chirality and nucleic acid structure influence complex formation, we examined turbidity profiles of poly-lysine variants (PLK_50_, PRK_50_, and PNK_50_) (Figure 1) with either dsDNA (≤2000 or 200–500 bp ranges) or tRNA across a range of charge fractions (Figure 2). We define a charge fraction ($f_{+}$) as the moles of our polycation over the total number of moles of polymer in our system (SI Charge Fraction Calculations, SI Equation (S3)). Turbidity measurements revealed a clear shift in the extent of phase separation across all nucleic acid systems as a function of charge fraction. Maximum turbidity was consistently recorded near the 0.5 nominal charge fraction across all systems, where roughly equal amounts of opposite charges were present in the solution, and electrostatic interactions were maximized.

Microscopy confirmed that for all three lysine variants, dsDNA yielded visible precipitation, indicative of solid-phase aggregation. In contrast, tRNA consistently promoted liquid–liquid phase separation (Figure 2 and Figures S2–S4). dsDNA samples formed irregular, dense precipitates regardless of polymer chirality, whereas tRNA produced more uniform coacervate droplets across all systems. These observations underscore the dominant role of nucleic acid secondary structure: the rigid double helix of long dsDNA favors interpolymer packing and precipitation, whereas the cloverleaf structure of tRNA allows hydration pockets and facilitates dynamic droplet formation. This is supported by previous studies showing that DNA complexes are biased towards precipitation unless the DNA strands are short [25] or the ionic strength of the solution is high due to salt addition [26]. Our observations with tRNA are also supported by previous work demonstrating that RNA complexation often occurs as coacervate droplets [27,28,29].

### 3.2. Complexation Extent and Morphology of Nucleotide Complexes

**Figure 3:**
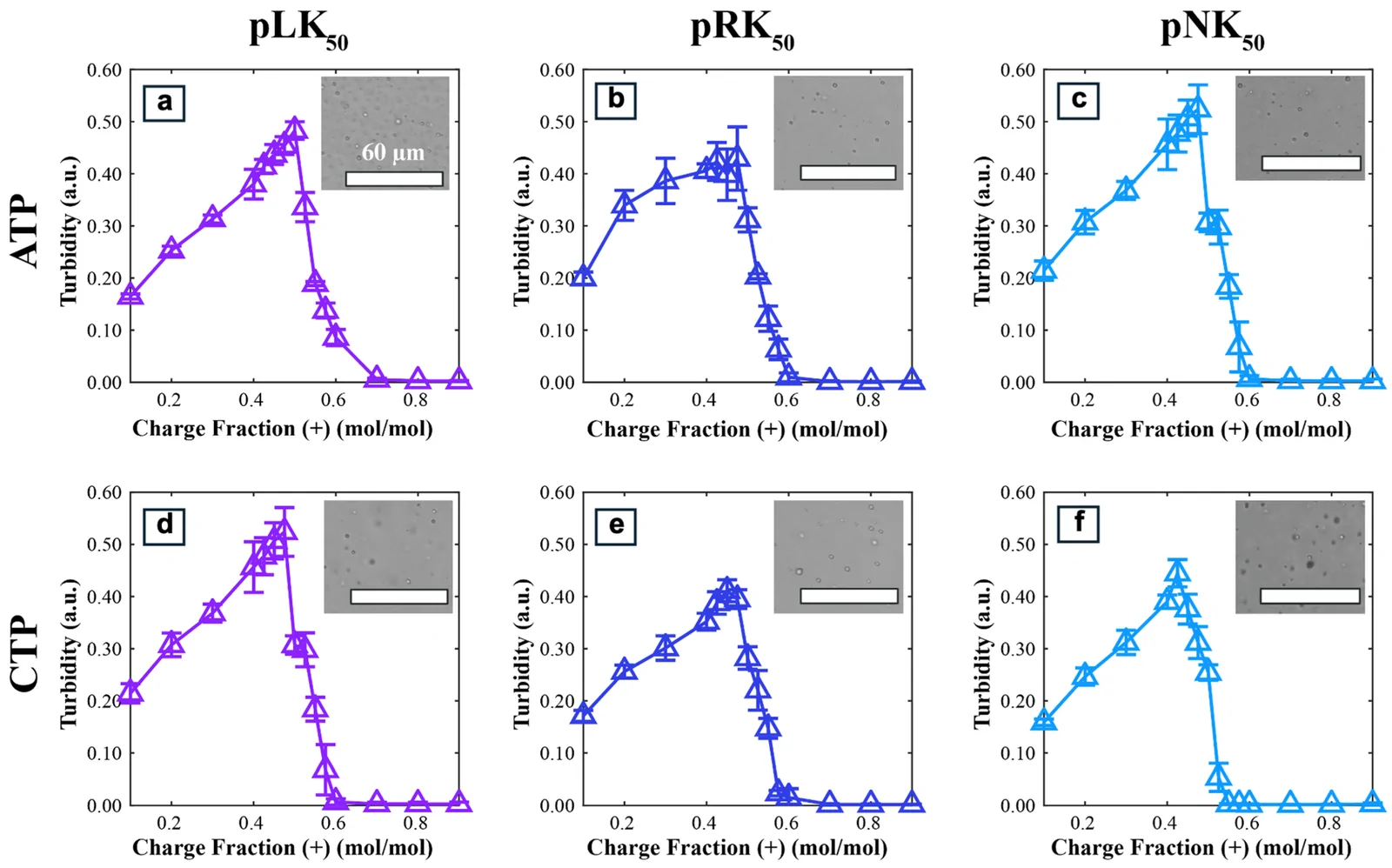
Turbidity measurements and micrographs of three poly-lysine variants (pLK_50_ in purple, pRK_50_ in dark blue, pNK_50_ in light blue) and nucleotides at varying polycation charge fractions. Turbidity versus charge fraction (+) (mol/mol) curves show the changes in turbidity readings and, consequently, the complexation. Micrographs were taken at each system’s respective peak turbidity. (**a**) ATP+PLK_50_, (**b**) ATP+PRK_50_, (**c**) ATP+PNK_50_, (**d**) CTP+PLK_50_, (**e**) CTP+PRK_50_, and (**f**) CTP+PNK_50_. Scale bars represent 60 µm in all micrographs.

We extended these studies to include nucleotides as a proxy for the smallest subunit of either DNA or RNA. Specifically, we investigated how small changes in ring structure affect complexation across chiral variants of poly-lysine. Through a combination of stoichiometry experiments and microscopy (Figure 3), we note that, with no added salt, the complexation follows standard trends. Around a charge fraction of 0.5, stoichiometry results indicate the highest levels of complexation, and, in all cases, we observe coacervate formation rather than precipitates.

These observations complement other studies that showed that nucleotide-based complexes result in coacervation [30]. These small molecules lack the secondary structure found in nucleic acids, leading to a balance of forces that preferentially form liquid–liquid phase separation rather than the overwhelming convergence of interactions with dsDNA that leads to precipitation.

### 3.3. Salt Concentration and Backbone Alignment Controlling Phase Separation of Nucleic Acid Complexes

**Figure 4:**
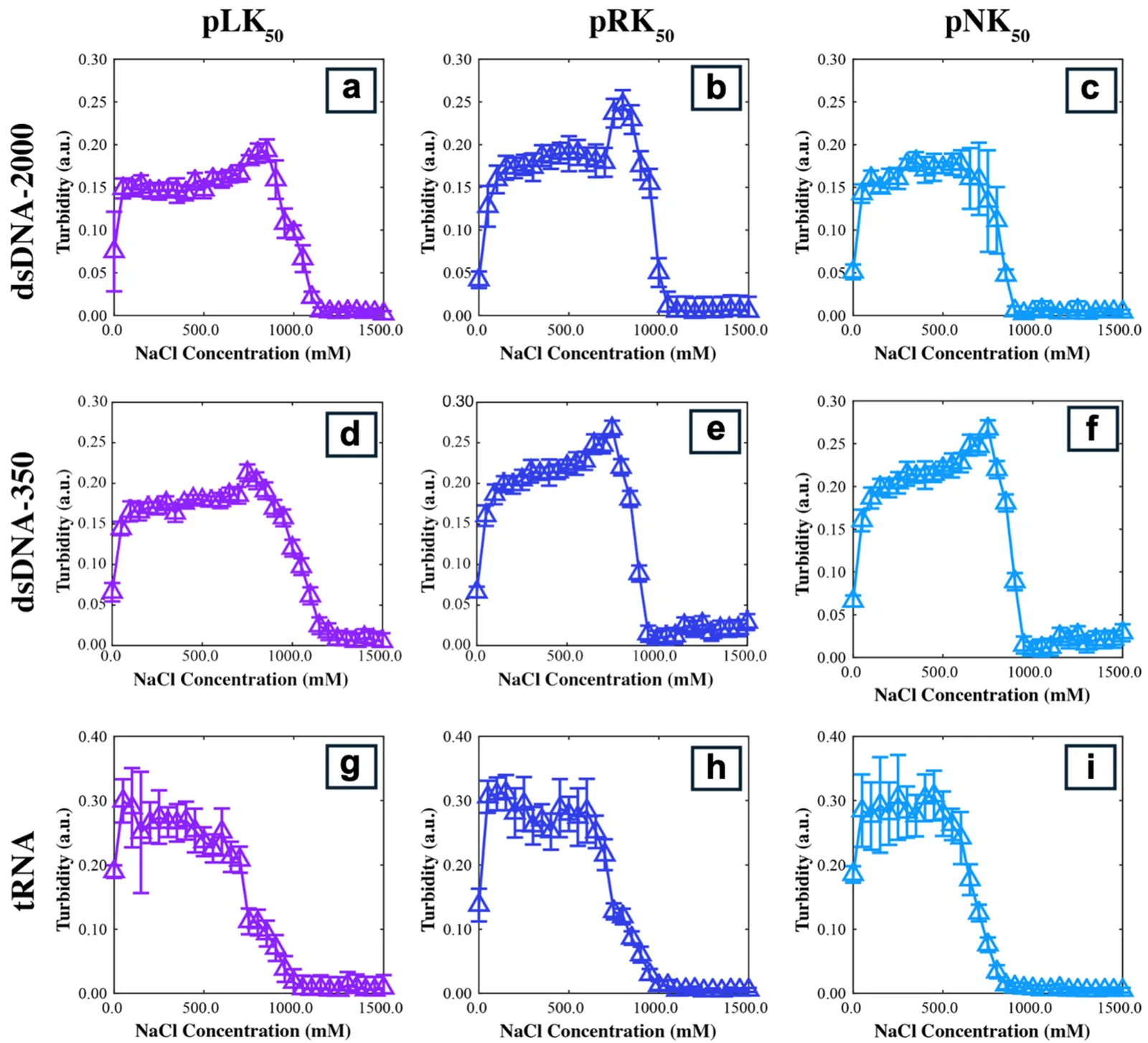
Turbidity measurements of three poly-lysine variants (pLK_50_ in purple, pRK_50_ in dark blue, pNK_50_ in light blue) and various nucleic acid combinations at varying NaCl concentrations at a mole fraction of 0.5. Turbidity versus NaCl concentration (mM) curves show an initial increase in phase separation and, consequently, in the turbidity reading, followed by a rapid decrease in turbidity as the concentration of added NaCl approaches the critical salt concentration for each system, at which phase separation is no longer observed. (**a**) dsDNA-2000+PLK_50_, (**b**) dsDNA-2000+PRK_50_, (**c**) dsDNA-2000+PNK_50_, (**d**) dsDNA-350+PLK_50_, (**e**) dsDNA-350+PRK_50_, (**f**) dsDNA-350+PNK_50_, (**g**) tRNA+PLK_50_, (**h**) tRNA+PRK_50_, and (**i**) tRNA+PNK_50_. Note that dsDNA 200–500 dp is denoted as dsDNA-350, and dsDNA with dp ≤ 2000 is denoted as dsDNA-2000.

We next investigated how ionic strength affects complex stability by gradually increasing the NaCl concentration in mixtures fixed at a charge fraction of 0.5 (Figure 4). In all nucleic acid systems, turbidity initially increased at low-to-moderate salt concentrations. This increase should not be interpreted simply as stronger electrostatic association, because salt screens rather than strengthens interactions between oppositely charged species. Instead, partial screening can weaken highly localized ionic contacts, increase chain mobility, and allow kinetically constrained complexes to rearrange, hydrate, or form larger light-scattering structures. Thus, the initial increase in turbidity may reflect changes in complex size, number, hydration, or morphology rather than an increase in binding strength alone [15,31].

At higher NaCl concentrations, sodium and chlorine ions increasingly screen the attraction between the cationic polymer side chains and nucleic acid phosphate groups, reducing the number and lifetime of polymer–nucleic acid ionic contacts. Increasing the bulk salt concentration also decreases the entropic benefit of releasing condensed counterions during complexation. Consequently, the complex phase becomes more hydrated and less polymer-rich as the system approaches its critical salt concentration. Above a system-specific critical salt resistance, electrostatic correlations are no longer sufficient to offset the entropy of mixing, the homogeneous phase becomes thermodynamically favored, and the complexes dissolve. The critical salt concentration therefore reflects the combined strength of electrostatic and non-electrostatic interactions within each polymer–nucleic acid pair [31,32,33]. In other words, beyond the system-specific critical salt concentration, polymer–solution interactions are more favorable than polymer–polymer interactions, leading to the dissolution of the complexes at high salt concentrations [34].

**Figure 5:**
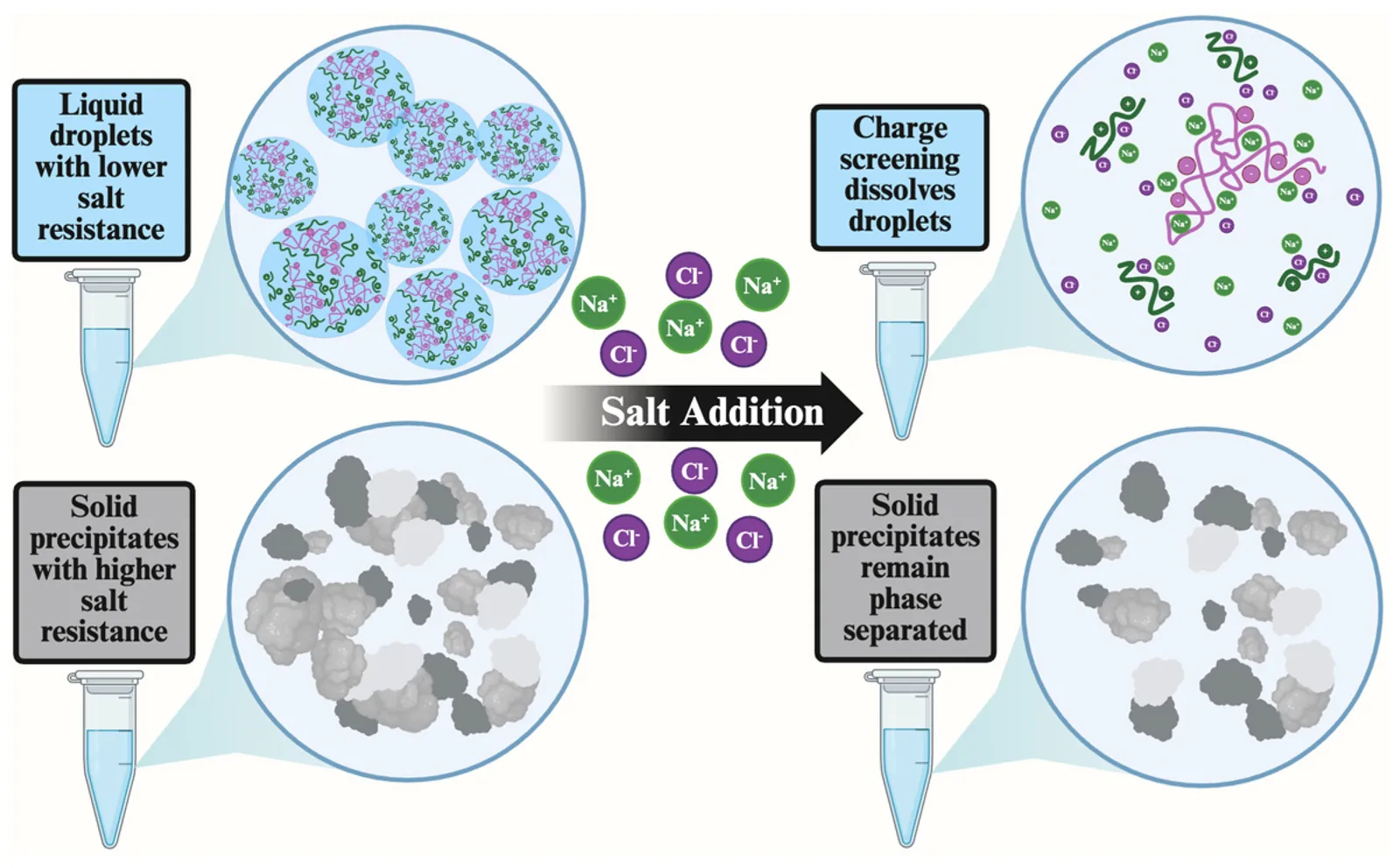
Schematic of the effects of salt addition on the morphological transitions complexes formed with poly-lysine variants and nucleic acids at each system’s respective peak turbidity reading. tRNA–polymer coacervate droplets dissolve upon reaching a transition salt concentration, and the solution further transitions back into a single phase. However, the critical salt concentration for dsDNA-polymer precipitates is higher, meaning these complexes resist redissolution into solution more effectively than coacervate droplets (Created in BioRender. Mirlohi, K. (2026) https://BioRender.com/tef9yyr, accessed on 2 September 2026).

Salt resistance studies were conducted over 0–1500 mM of added NaCl. Across both dsDNA lengths, precipitates formed well beyond the biologically relevant salt concentration. In contrast, tRNA–polymer droplets began to undergo phase transitions, shifting into aggregates at lower salt concentrations but resisting dissolution back into a single-phase state until higher salt concentrations, highlighting the lower salt resilience of coacervate droplets and the higher salt resilience of aggregates formed with tRNA (Figure 5). Representative micrograph series of morphology transitions confirm this distinction (Figures S3–S5). Although the range of salt concentrations far exceeds that relevant to biological systems, it provides insight into whether changes in chirality can alter salt stability. Specifically, we see subtle shifts in the critical salt concentration as we move from a homochiral to mixed-chiral to achiral poly-lysine. With dsDNA, we observe a larger shift in this salt resistance as the system is complexed with pLK_50_ to pRK_50_, with a smaller decrease in the critical salt concentration from our racemic poly-lysine to the peptoid system. A similar trend is observed with tRNA, but with more subtle shifts to the left as the poly-lysine chirality changes from homochiral to racemic to achiral.

### 3.4. Salt Concentration and Ring Composition Controlling Phase Separation of Nucleotide Complexes

**Figure 6:**
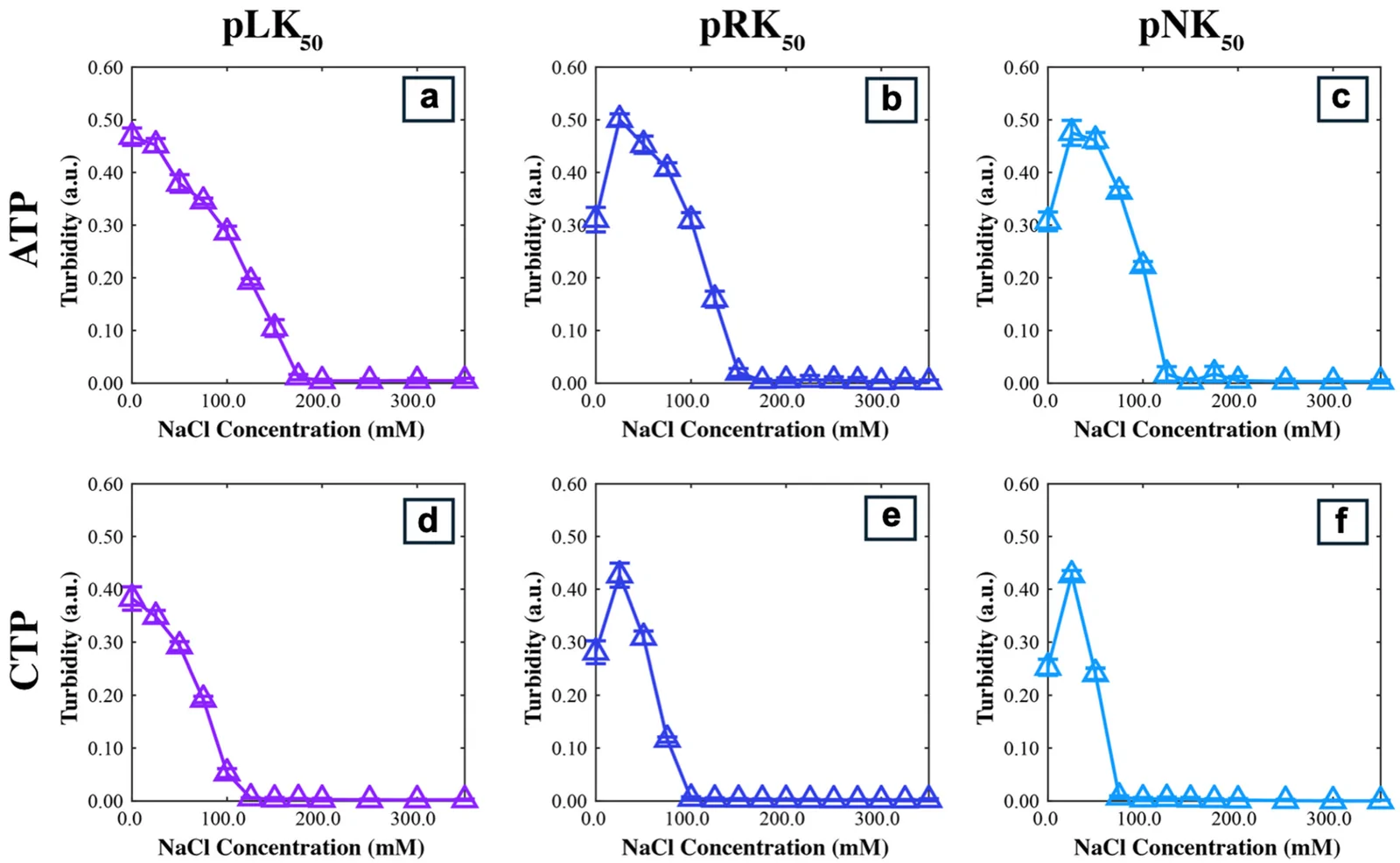
Turbidity measurements of three poly-lysine variants (pLK_50_ in purple, pRK_50_ in dark blue, pNK_50_ in light blue) and nucleotide combinations at varying NaCl concentrations at a mole fraction of 0.5. Turbidity versus NaCl concentration (mM) curves show an initial increase in phase separation for mixed or achiral lysine systems and, consequently, in the turbidity reading, followed by a rapid decrease in turbidity as the concentration of added NaCl approaches the critical salt concentration for each system, at which phase separation is no longer observed. (**a**) ATP+PLK_50_, (**b**) ATP+PRK_50_, (**c**) ATP+PNK_50_, (**d**) CTP+PLK_50_, (**e**) CTP+PRK_50_, and (**f**) CTP+PNK_50_.

To complement our salt studies with nucleic acids, we next investigated how increasing NaCl concentration affects the stability of our lysine-nucleotide complexes at a constant charge fraction of 0.5. As with DNA and RNA, our lysine complexes show turbidity shifts as a function of chirality (Figure 6). From homochiral to mixed-chiral to achiral lysine polymer systems, we observe turbidity readings reaching background at lower salt concentrations. Furthermore, we see a shift in the NaCl concentration between ATP and CTP with the same lysine polymer, where CTP systems consistently reach zero turbidity before ATP systems.

### 3.5. Morphology as a Function of Salt

As suggested by Figure 2, distinct morphologies were observed between our dsDNA and tRNA systems. When complexation occurred with dsDNA, we observed precipitation of larger aggregates at lower salt concentrations. As the salt concentration increased, these aggregates became smaller and more dispersed. When we compare the microscopy (Figures S3–S5) with the salt curves (Figure 4), we note that each system saw an increase in turbidity reading, corresponding to an increase in the number of complexes. With dsDNA, we observe denser aggregation of precipitates that continues until approximately 800 mM NaCl, before salt resistance, where smaller precipitates dominate. The sharp decrease in the salt curves coincides with sparser aggregates forming prior to the critical salt curve.

The same experiments with tRNA led to an initial increase in droplets and wetting of the bottom of the well plate. Interestingly, with the achiral PNK_50_ system, we observed an almost melt-like morphology at low salt concentration. As the systems approach their critical salt concentrations, we observe less wetting and smaller coacervate droplets.

In the case of our nucleotides, they follow a similar pattern to our dsDNA systems, where we observe no transition state from the polyelectrolyte complexes to a single-phase solution. However, instead of precipitates, coacervates are formed until the critical salt concentration is reached (Figures S6 and S7).

### 3.6. The Phase Space of Poly-lysines with Nucleic Acids and Nucleotides

**Figure 7:**
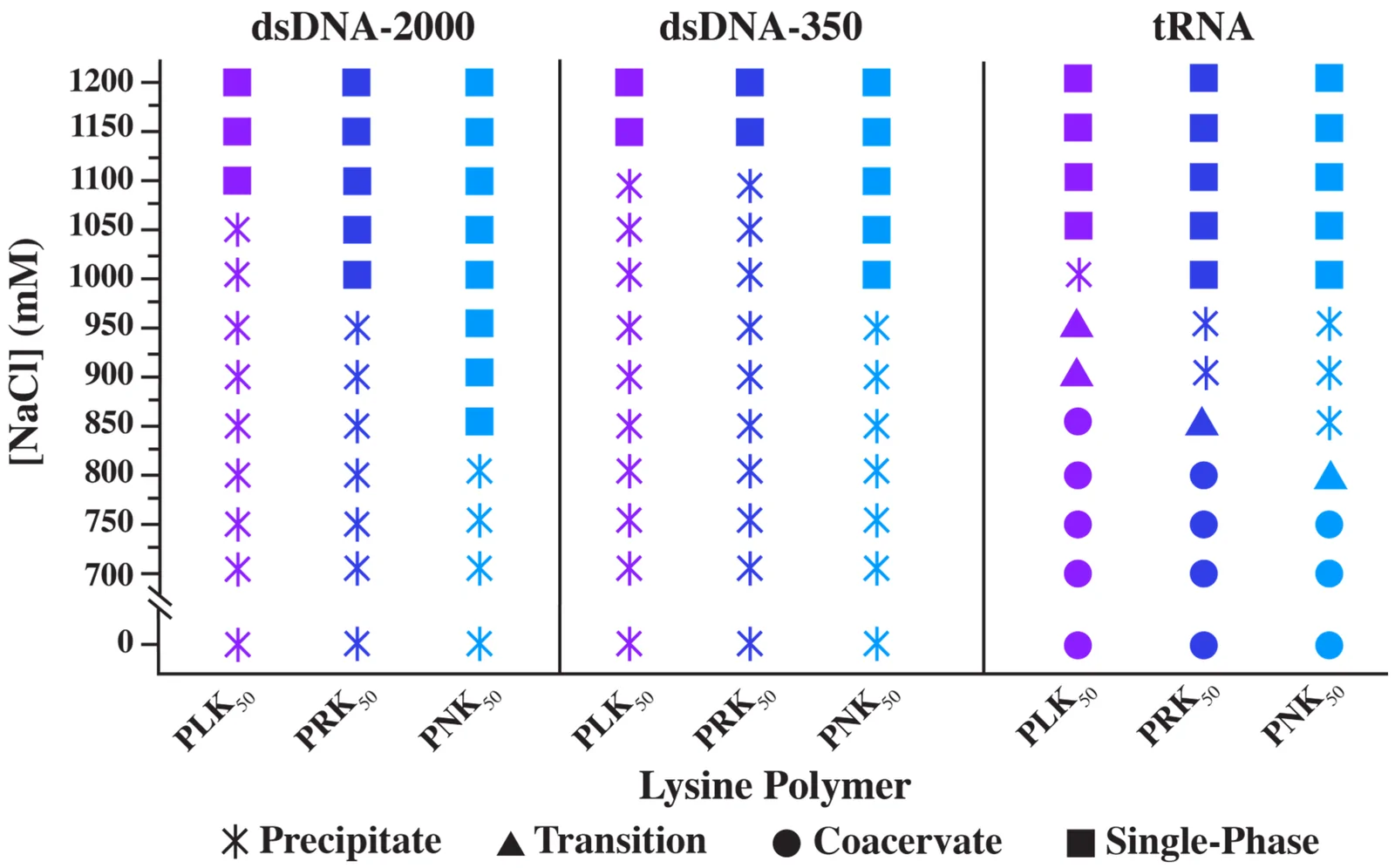
Phase chart of all nucleic acid–polymer (pLK_50_ in purple, pRK_50_ in dark blue, pNK_50_ in light blue) combinations at various salt concentrations at 0.5 mole fraction. In all dsDNA systems, complexes transitioned from precipitates to a single-phase state, whereas in all tRNA systems, complexes transitioned from coacervate droplets to precipitates first, before the phase separation disappeared. Additionally, the results make it evident that complexes formed with the homochiral polymer exhibit greater salt resistance than those involving the racemic species or the polypeptoid, highlighting the important role of backbone alignment in complexation. Note that dsDNA 200–500 dp is denoted as dsDNA-350, and dsDNA with dp ≤ 2000 is denoted as dsDNA-2000.

**Figure 8:**
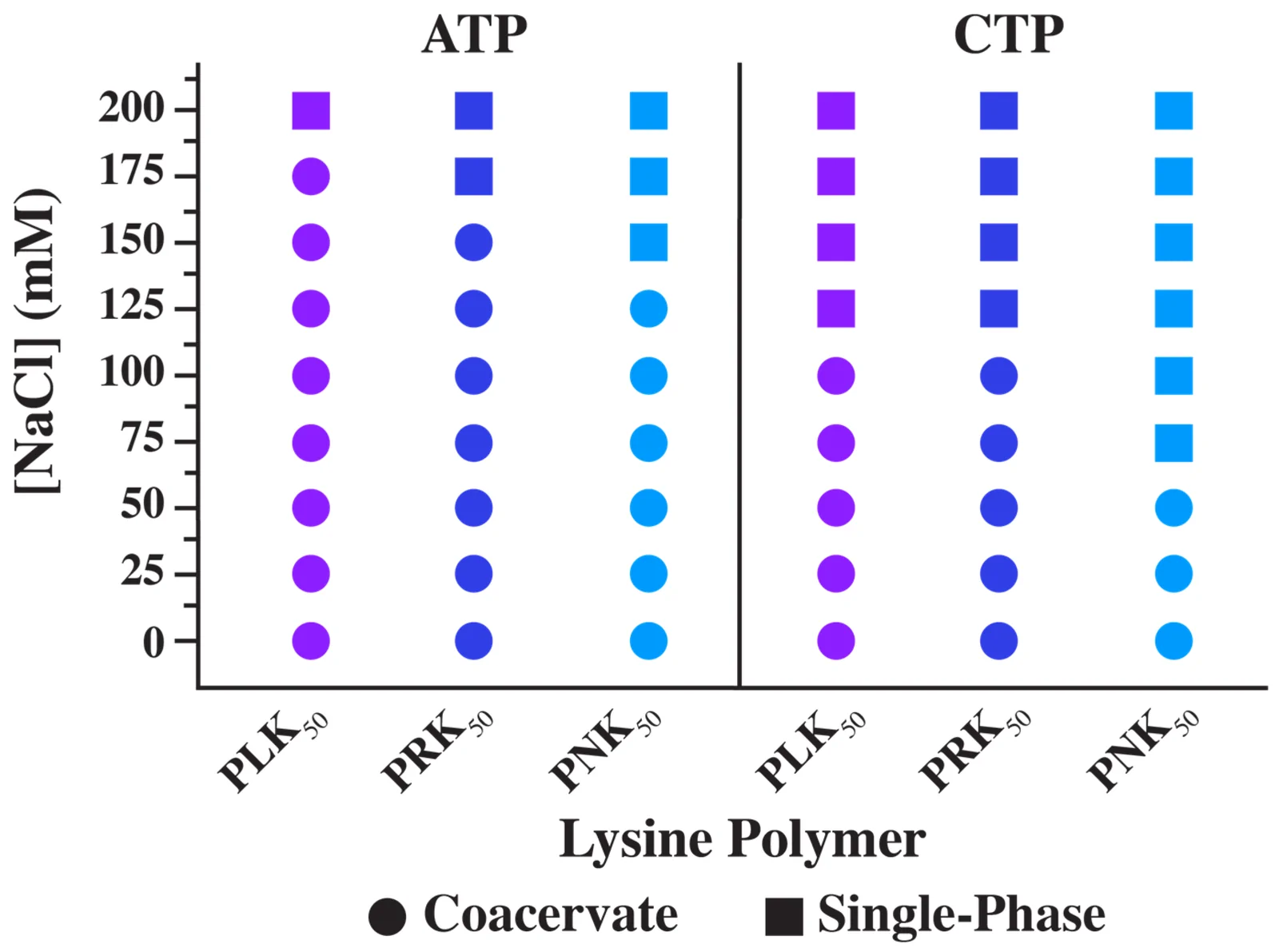
Phase chart of all nucleotide–polymer (pLK_50_ in purple, pRK_50_ in dark blue, pNK_50_ in light blue) combinations at various salt concentrations at 0.5 mole fraction. In all ATP and CTP systems, complexes formed coacervates and transitioned directly to a single-phase solution without an intermediate phase.

If we collect all microscopy data, we can form phase diagrams for all systems as salt concentration increases (Figure 7 and Figure 8). We observe that as we move from homochiral systems to mixed-chiral to achiral, not only does the stability or salt resistance (Table 1) decrease, but the window for a transition (if present) decreases. Interestingly, for the tRNA systems, as the window for transitional morphology decreases, the window for precipitation widens. 

These trends continue with our nucleotide systems, where the chirality of the backbone plays a subtle role in the salt resistance. As we move from a homochiral to a mixed-chiral and finally achiral lysine polymer, we see a decrease in the critical salt concentration (Figure 8). Furthermore, as the size of the nucleotide decreases from ATP (double ring) to CTP (single ring), we see additional influences on the salt resistance, with the required salt concentration to achieve a single-phase solution decreasing. It is interesting to note that despite the higher charge concentration observed in ATP and CTP, we see a remarkably lower salt resistance than our dsDNA and tRNA systems. We attribute this to the lack of secondary and higher-level structures present in our nucleic acid systems, which allow multivalent interactions.

## 4. Discussion

### 4.1. Complexation with Nucleic Acids and Nucleotides

dsDNA drives precipitation due to its conformational rigidity and higher charge density compared to tRNA, thereby strengthening electrostatic interactions with cationic polymers, whereas tRNA promotes coacervation by enabling more flexible, liquid-like associations. Across all chiralities, we observed the same type of complexation, indicating that electrostatics and nucleic acid structure are the dominant forces governing complexation in the absence of salt.

In this sense, chirality plays a less important role in dictating the type of complexation in the absence of salt. Thus, when selecting lysine-based polymers for biomedical applications, achiral poly-N-lysine has great potential for forming complexes with nucleic acids.

For the nucleotide systems, we note that all systems preferentially formed coacervate droplets when complexation occurred as a function of charge fraction. Interestingly, all systems formed coacervates at low poly-lysine charge fraction ($f_{+}<0.5$), suggesting that these small molecules can complex when in abundance and in the presence of a polycation.

### 4.2. Salt Stability Across Chirality

A consistent trend across salt studies was the enhanced stability of systems with some form of chirality, with L-homochiral poly-lysine (PLK_50_) complexes being the most stable against salt addition, followed by PRK_50_ complexes, and PNK_50_ complexes the least stable (Figure 4). These observations suggest that backbone alignment in the homochiral polymer enhances favorable electrostatic interactions and maintains network integrity under ionic stress, and some level of chirality, such as in PRK_50_, increases stability at higher ionic strengths, even if not perfectly ordered as in a homochiral polymer. In contrast, the N-substituted backbone of PNK_50_ exhibited reduced stability, dissolving at lower salt concentrations and producing less sharply defined turbidity transitions.

Although the three polycations contain similar cationic side chains, their backbone structures affect how these charges are spatially presented and how efficiently the chains can pack and interact with their environment and other charged species. PLK has a stereoregular polypeptide backbone capable of backbone hydrogen bonding, whereas the mixed D and L sequence of PRK disrupts regular packing; PNK contains N-substituted tertiary amides and lacks both backbone hydrogen-bond donors and α-carbon chirality, resulting in greater conformational heterogeneity [35,36]. These differences likely reduce cooperative packing in PNK complexes and account for their lower tendency to form compact precipitates. Nucleic acid architecture remains the dominant influence on morphology, with rigid, highly charged dsDNA favoring interchain bridging and precipitation, while the compact and branched structure of tRNA favors more dynamic coacervate droplets [37]. Taken together, these results demonstrate that phase behavior in polymer–nucleic acid complexes is jointly determined by nucleic acid secondary structure and polymer chirality (Table 1).

In addition to the effects of nucleic acid secondary structure, it is also evident that homochiral polymers further stabilize nucleic acid complexes against salt addition (Figure 4, Figure 7 and Figures S3–S5). Interestingly, the results show a visual difference in the response of the two systems to salt addition (Figure 7 and Figures S3 and S4): while dsDNA complexes dissolve at each system’s respective critical salt concentration and the solution returns to a single phase, tRNA complexes transition from coacervate droplets into precipitates first, before they dissolve, and the phase separation disappears (Figure 7 and Figure S5). This can be explained by the fact that dsDNA has a structure close to that of a rigid rod, lacking the flexibility of tRNA complexes, which leads to a wider variety of morphologies in solution. Additionally, the introduction of salt may promote chain rearrangement and allow instances of double-strandedness in tRNA through base stacking and hydrogen bonding [38,39]. These observations emerge as key factors in complexation formulation.

However, in terms of biological relevance, all three lysine polymers, whether complexed with dsDNA or tRNA, exhibited salt resistance above physiological levels. From this standpoint, all three lysine polymers can maintain complexes at 150 mM NaCl.

With the nucleotide systems, we observed much lower salt resistance, consistent with previous work indicating that the smaller polyelectrolyte length dominates the critical salt concentration [40], a macromolecular connectivity effect. The chirality followed the same patterns in salt resistance as our dsDNA and tRNA systems, indicating subtle influences of chirality. Interestingly, the number of rings in the nucleotide plays a nuanced role in the critical salt concentration. In ATP, where there is a double ring, we see a higher salt resistance, while CTP with a single ring has a lower salt resistance. This may be attributed to the number of hydrogen-bonding sites, as the purine ring has more sites than the pyrimidine ring. Spruijt and co-workers showed similar results when correlating temperature stability back to salt stability for nucleotide-poly(L-lysine) systems [41]. In this work, they noted that the critical salt concentration as a function of temperature showed higher resistance for GTP and ATP than CTP, UTP, and TTP with poly(L-lysine). Although there were subtle differences between these two groups, the purine structures consistently displayed higher salt resistance than the pyrimidines. Additionally, this correlation between temperature and salt stability may provide further design rules for formulation by comparing salt resistances. This could help predict which sequences of nucleic acids will have relatively higher or lower critical salt concentrations based on the number of purines (double-ringed) and pyrimidines (single-ringed) when complexed with the same polycation.

## 5. Conclusions

This study demonstrates that the phase behavior and stability of nucleic acid– or nucleotide–polymer complexes in model systems are strongly governed by both the identity of the nucleic acid or nucleotide and the polymer chirality. While dsDNA consistently formed precipitates with lysine-based polypeptides and polypeptoids, tRNA supported the formation of liquid coacervate droplets, highlighting the role of secondary structure in guiding phase outcomes. Furthermore, the lack of secondary structure in nucleotides suggests that, without these strong interactions, coacervation is preferred.

Among the lysine polymer variants, the L-homochiral poly-lysine complexes exhibited the greatest stability under increasing salt concentrations, underscoring the importance of backbone alignment in maintaining electrostatic interactions at higher ionic strengths in all system sets. Additionally, dsDNA complexes dissolved from precipitates back into solution, returning to a single phase, whereas tRNA coacervate droplets transitioned into solid aggregates before dissolving, and phase separation disappeared. Interestingly, ATP and CTP exhibited features of both observations. The nucleotides formed coacervates akin to the tRNA systems but showed no transitional phase between the polyelectrolyte complexes and a single-phase solution, as in the dsDNA systems. This may be attributed to the lack of a backbone despite the higher charge density.

By systematically comparing stoichiometry and salt resistance across these systems, our findings establish clear links between polymer chirality, nucleic acid/nucleotide structure, and ionic strength in directing coacervation versus precipitation. These insights expand the fundamental understanding of nucleic acid complexation and provide a framework for the rational design of salt-tolerant, peptide-based encapsulation platforms. Although our model molecules differ from therapeutic cargoes in sequence, size, structure, and biological function, our results are most relevant to future delivery-system development as design rules for nucleic acid-based therapeutics, including mRNA, siRNA, antisense oligonucleotides, guide RNA, and plasmid DNA. Higher CSCs enable numerous applications, such as long-term delivery matrices, bioadhesives, and use in aquatic systems that must resist dissolution at high ionic strengths. Ultimately, this work contributes to ongoing efforts to engineer stable and efficient carriers for various applications, including nucleic acid therapeutics, where control over phase morphology and stability is essential for clinical translation.

## Figures and Tables

**Table 1:** Salt resistance and change in morphology of complexes for each nucleic acid/nucleotide–polymer system and the respective salt concentration. Nucleic acid/nucleotide–polymer complexes involving PLK_50_ generally show higher salt resistance compared to complexes formed with racemic or achiral poly-lysine variants. Changes in morphology, if observed, are noted in blue.

Change in Morphology (mM), Salt Resistance (mM)			
	PLK_50_	PRK_50_	PNK_50_
dsDNA-2000	1100	1000	850
dsDNA-350	1150	1150	1000
tRNA	900, 1050	850, 1000	800, 1000
ATP	200	175	150
CTP	125	125	75

## Data Availability

The original contributions presented in this study are included in the article and Supplementary Materials. Further inquiries can be directed to the corresponding author.

## Supplementary Materials

The following supporting information can be downloaded at: https://www.mdpi.com/article/10.3390/biom16091315/s1, HPLC Analysis; Figure S1: Representative HPLC chromatograms of PLK (top), PRK (middle), PNK polymer (bottom); Figure S2: Coalescence of coacervate droplets in tRNA and PRK_50_ as a representative system; Figure S3: Micrographs of dsDNA-2000 with (a) PLK50, (b) PRK50, and (c) PNK50 complexes (charge fraction 0.5) at varying concentrations of 0, 50, 700, 950, 1050, and 1150 mM NaCl, highlighting morphology transitions; Figure S4: Micrographs of dsDNA-350 with (a) PLK50, (b) PRK50, and (c) PNK50 complexes (charge fraction 0.5) at varying concentrations of 0, 50, 700, 950, 1050, and 1150 mM NaCl, highlighting morphology transitions; Figure S5: Micrographs of tRNA with (a) PLK50, (b) PRK50, and (c) PNK50 complexes (charge fraction 0.5) at varying concentrations of 0, 50, 700, 950, 1050, and 1150 mM NaCl, highlighting morphology transitions; Figure S6: Micrographs of ATP with (a) PLK50, (b) PRK50, and (c) PNK50 complexes (charge fraction 0.5) at varying concentrations of 0, 25, 50, 100, 150, and 200 mM NaCl, highlighting morphology transitions; Figure S7: Micrographs of CTP with (a) PLK50, (b) PRK50, and (c) PNK50 complexes (charge fraction 0.5) at varying concentrations of 0, 25, 50, 100, 150, and 200 mM NaCl, highlighting morphology transitions; Table S1: Nucleotides and their Respective Molecular Weights; Nucleic Acid Molecular Weight Calculations; Charge Fraction Calculations; References [42,43,44] are cited in the Supplementary Materials.

## Abbreviations

The following abbreviations are used in this manuscript:

a.u.	Arbitrary units
dsDNA	Double-stranded deoxyribonucleic acid
LLPS	Liquid–liquid phase separation
PLK	Poly(L-lysine)
PNK	N-substituted poly(lysine)
PRK	Poly(D,L-lysine)
tRNA	Transfer ribonucleic acid
ATP	Adenosine Triphosphate
CTP	Cytidine Triphosphate
