# Supplementary material for: Complexation of Polypeptides and Polypeptoids with Nucleic Acids: Does Chirality Matter for Salt Stability and Morphology?
Source: Biomolecules. 2026 Sep 10;16(9):1315. doi: 10.3390/biom16091315 (PMC13604238; doi:10.3390/biom16091315)
Supplement: Supplementary file 1 [file biomolecules-16-01315-s001.zip › biomolecules-4511367-supplementary.pdf]

Reverse-Phase High performance liquid chromatography (RP-HPLC)

RP-HPLC analysis was performed with Thermo scientific Vanquish system, which includes Vanquish Pump, Vanquish Autosampler, Vanquish Column Compartment, and Vanquish Diode Array Detector. Chromeleon 7.3.1 software was used to control the system and process data. The sample was separated on a Waters XSelect CSH C18 column (5um, 4.6 x 250 mm) with XSelect CSH C18 guard column (5 um, 4.6 x 20 mm) at room temperature. The mobile phase consisted of eluent A (0.1% TFA in water) and eluent B (0.1% TFA in acetonitrile). The linear gradient start at A:B= 95:5 and linear to A:B=45:55 in 50 min and linear to A:B=5:95 in 5 min. Then the column was cleaned with A:B = 5:95 for 5 minutes and equivalent to A:B=5:95 for 10 minutes before next injection. The flow rate is 1.0 ml/min. The detected wavelength was set at 215 nm. Samples were prepared for analysis by dissolving the polymers (1 mg/mL) in a 95% water/0.1% TFA/5% acetonitrile solvent mixture. Injection volume is 20 ml.

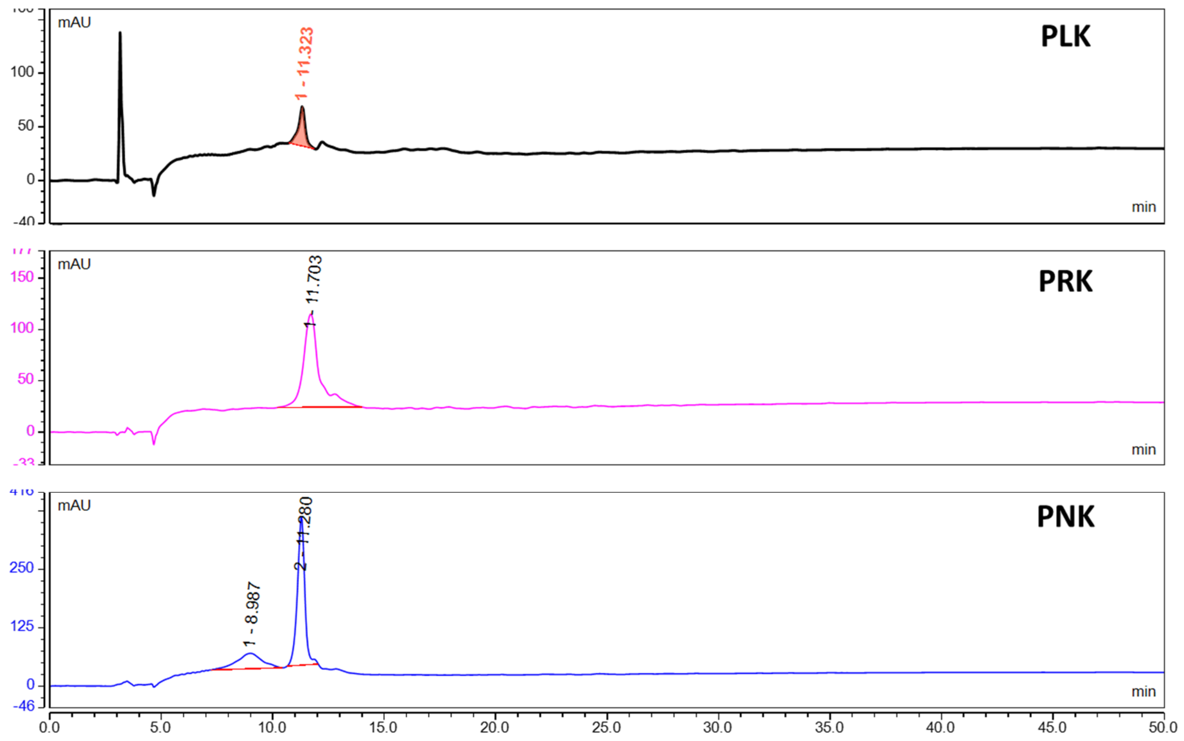

Figure S1. Representative HPLC chromatograms of PLK (top), PRK (middle), PNK polymer (bottom). All three polymers eluted at about 11 min, suggesting no significant differences in hydrophobicity among the samples.

Nucleic Acid Molecular Weight Calculations

To estimate the molecular weight (MW) of our nucleic acids, we assumed an average nucleotide composition of ~25% for each of the four bases (A, U/T, G, and C).[1,2] This approximation is supported by available sequence databases (e.g., GtRNAdb for S. *cerevisiae* tRNAs),[3] which show that despite the heterogeneity of individual sequences and isoacceptor families (Table 1), the overall monomer composition converges to ~339-341 g/mol per nucleotide. While post-transcriptional modifications and sequence variation introduce some uncertainty, these effects average out across the diverse nucleic acid populations present in our samples, making the 25% assumption both practical and scientifically sound for these model systems. We purchased 10 mg/mL solutions of nucleic acids from

Thermo Fisher Scientific, which were at ~30 mM concentration (Equations 1 and 2). We then diluted these samples to 10 mM using autoclaved, ultrapure water for experiments.

Importantly, our empirical results validate this approach: across all systems and nucleic acid types studied, we consistently observed peak turbidity near a 0.5 charge fraction, which means that's where electrostatic interactions were optimized, and therefore, the charges in the 10 mM solutions of the nucleic acids are balanced with the charges of the oppositely charged polycations at 10 mM. This alignment between theoretical stoichiometry and experimental outcome indicates that our MW estimations, and the concentrations derived from them, were appropriate and accurate.

Table S1. Nucleotides and their respective molecular weights.[2].

Nucleotide	MW (g/mol)
A	331.2
T	322.2
U	324.2
G	347.2
C	307.2

$$\boldsymbol{Average\ MW} = \frac{\mathbf{331.2+322.2+347.2+307.2}}{\mathbf{4}} = \mathbf{327.0} \approx \mathbf{330}\ \left(\frac{\boldsymbol{g}}{\boldsymbol{mol}}\right) \quad \text{(Equation S1)}$$

$$\boldsymbol{Concentration} = \frac{\mathbf{0.010}\left(\frac{\boldsymbol{g}}{\boldsymbol{mL}}\right)}{\mathbf{330}\left(\frac{\boldsymbol{g}}{\boldsymbol{mol}}\right)} = \mathbf{3.03} * \mathbf{10^{-5}}\left(\frac{\boldsymbol{mol}}{\boldsymbol{mL}}\right) \approx \mathbf{30}\ \boldsymbol{mM} \quad \text{(Equation S2)}$$

Charge Fraction Calculations

In our study, charge fraction (f_+) represents the fraction of total charge that is positive, calculated on a monomer (charge) basis. Because all concentrations are defined per monomer (i.e., per charge), f_+ directly reflects the proportion of positive charges relative to the total charge in the mixture:

$$f_+ = \frac{[+]}{[+]+[-]} \quad \text{(Equation S3)}$$

Here, [+] and [−] are the molar concentrations of polymeric cationic and nucleic-acid or nucleotide anionic monomers, respectively. Thus:

- f_+ = 0.5 corresponds to nominal charge stoichiometry (equal positive and negative charges)
- f_+ < 0.5 indicates nucleic-acid or nucleotide excess
- f_+ > 0.5 indicates polymer excess

For example, a total monomer concentration of 2 mM refers to 2 mM of total charge sites (polymer + nucleic acid or nucleotide). Lastly, the maximum turbidity occurring consistently near $f_+ \approx 0.5$ supports this definition.

Coalescence Observations

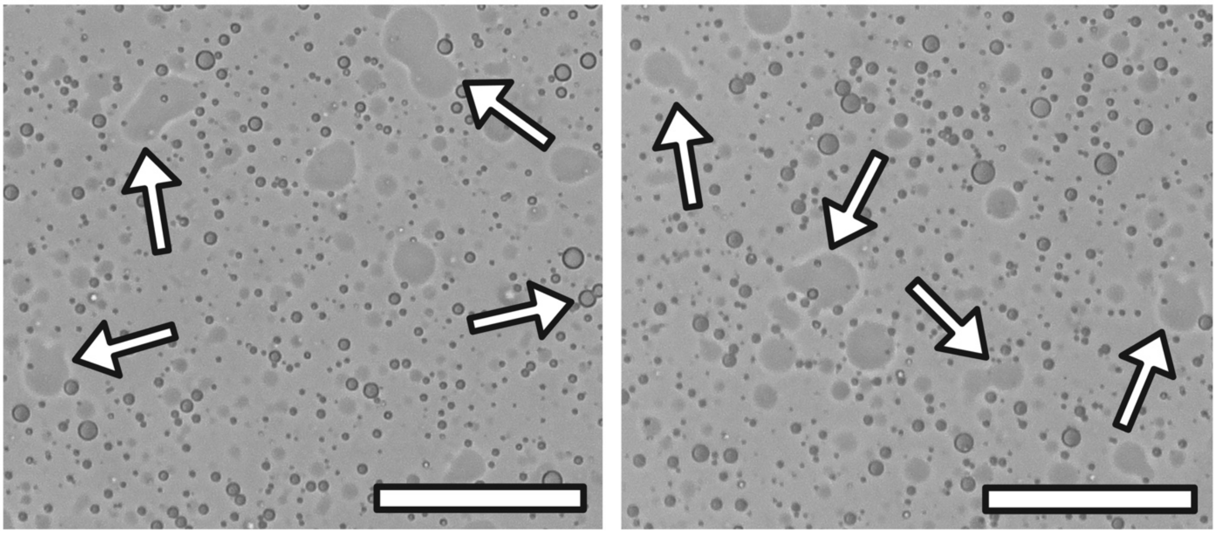

Figure S2. Coalescence of coacervate droplets in tRNA and PRK_{50} as a representative system. The liquidity of droplets can be verified by their tendency to coalesce, a hallmark characteristic of bulk coacervate droplets. Scale bars represent 60 μm.

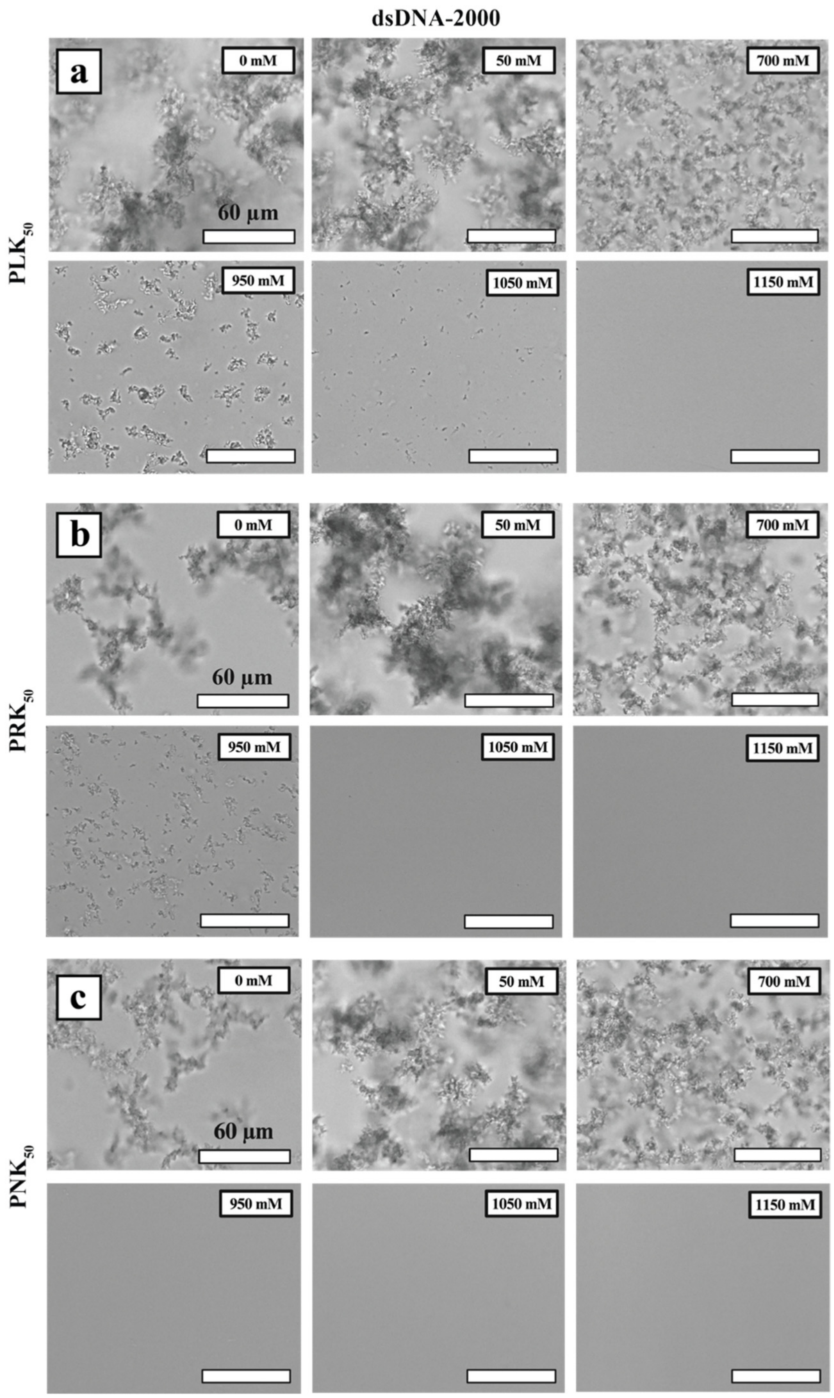

Figure S3. Micrographs of dsDNA-2000 with **(a)** PLK_{50}, **(b)** PRK_{50}, and **(c)** PNK_{50} complexes (charge fraction 0.5) at varying concentrations of 0, 50, 700, 950, 1050, and 1150 mM NaCl, highlighting morphology transitions. PLK_{50} complexes were more resistant to salt addition compared to PNK_{50} and PRK_{50} complexes. Scale bar = 60 microns in all micrographs.

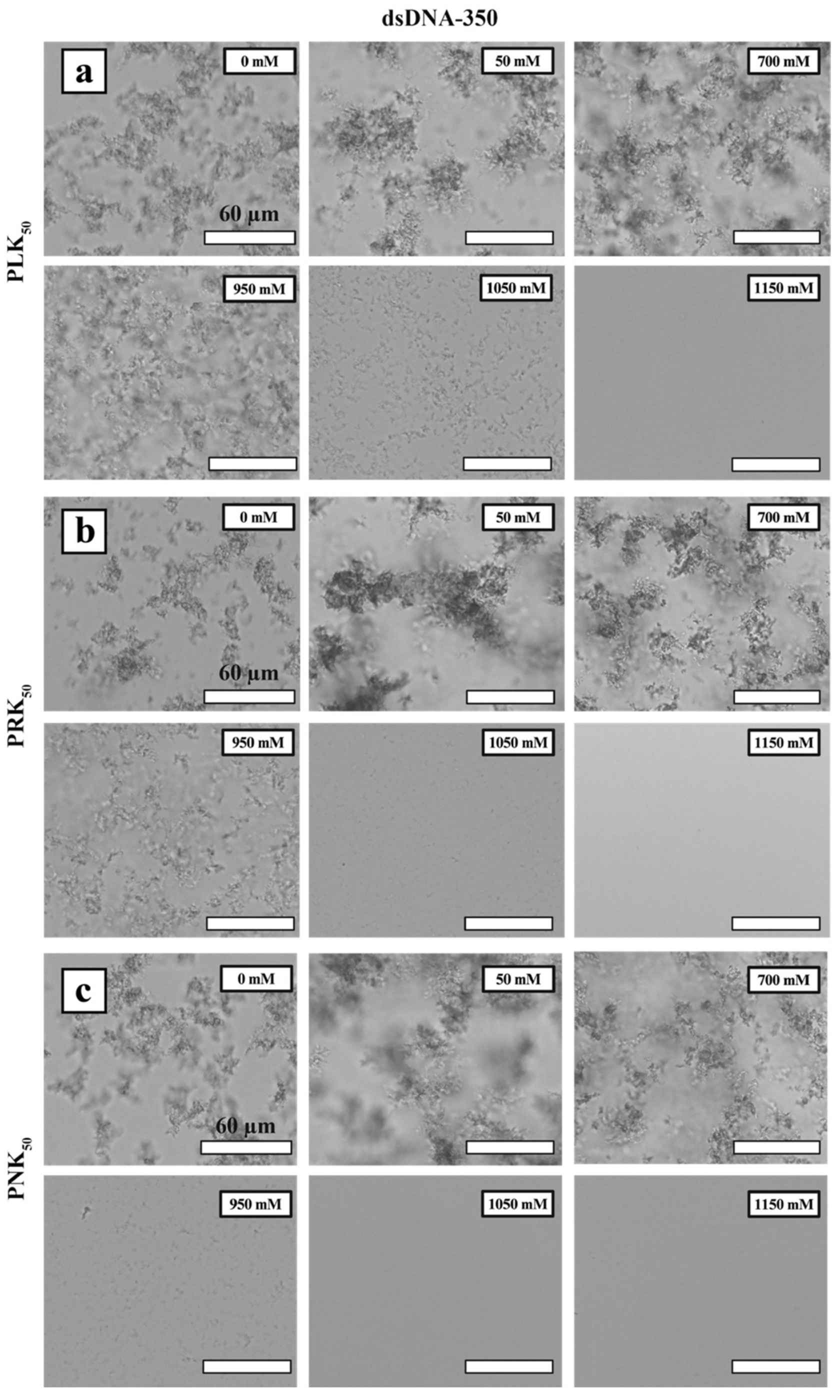

Figure S4. Micrographs of dsDNA-350 with **(a)** PLK_{50}, **(b)** PRK_{50}, and **(c)** PNK_{50} complexes (charge fraction 0.5) at varying concentrations of 0, 50, 700, 950, 1050, and 1150 mM NaCl, highlighting morphology transitions. PLK_{50} and PRK_{50} complexes were more resistant to salt addition compared to PNK_{50} complexes. Scale bar = 60 microns in all micrographs.

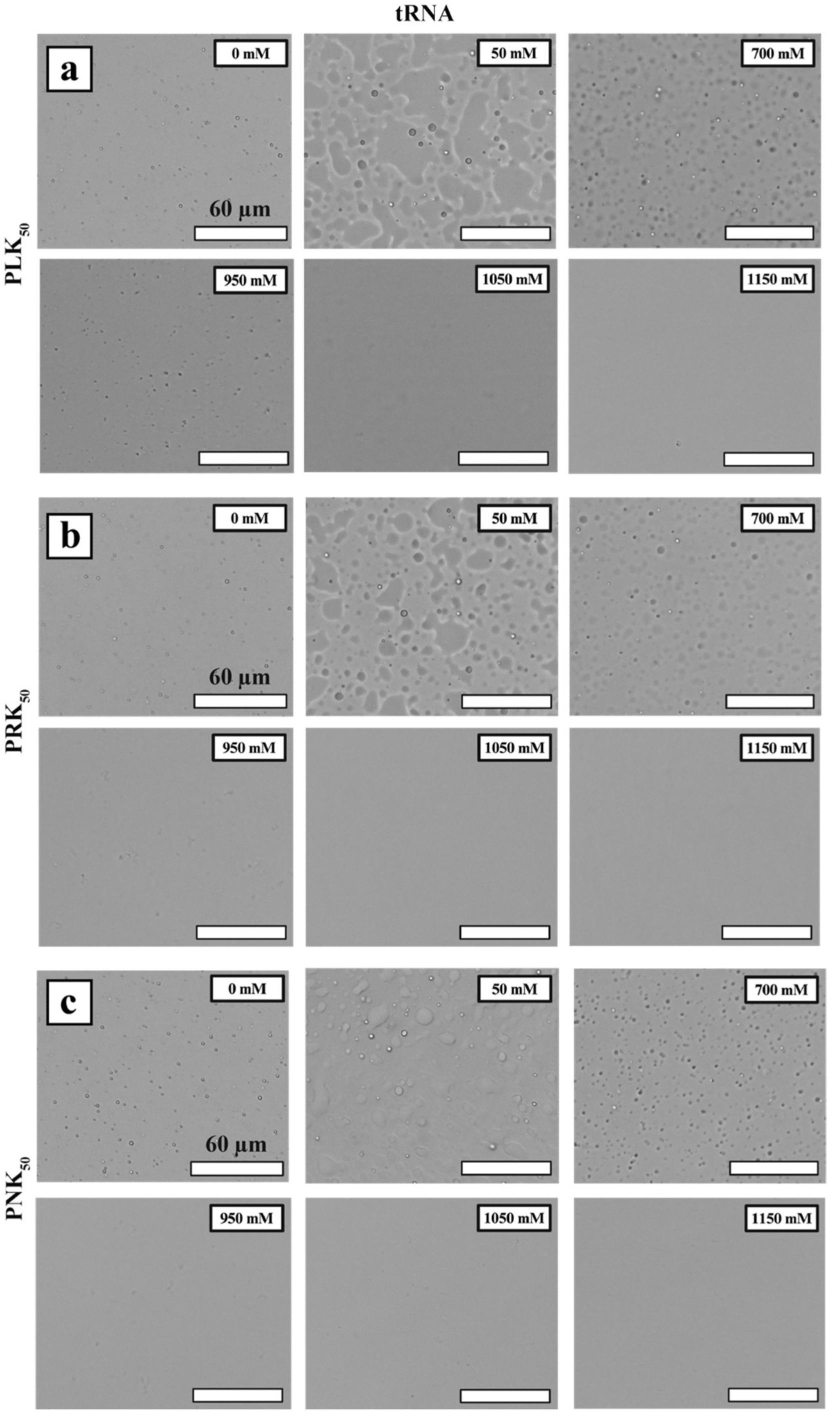

Figure S5. Micrographs of tRNA with **(a)** PLK_{50}, **(b)** PRK_{50}, and **(c)** PNK_{50} complexes (charge fraction 0.5) at varying concentrations of 0, 50, 700, 950, 1050, and 1150 mM NaCl, highlighting morphology transitions. PLK_{50} complexes were more resistant to salt addition compared to PNK_{50} and PRK_{50} complexes. Scale bar = 60 microns in all micrographs.

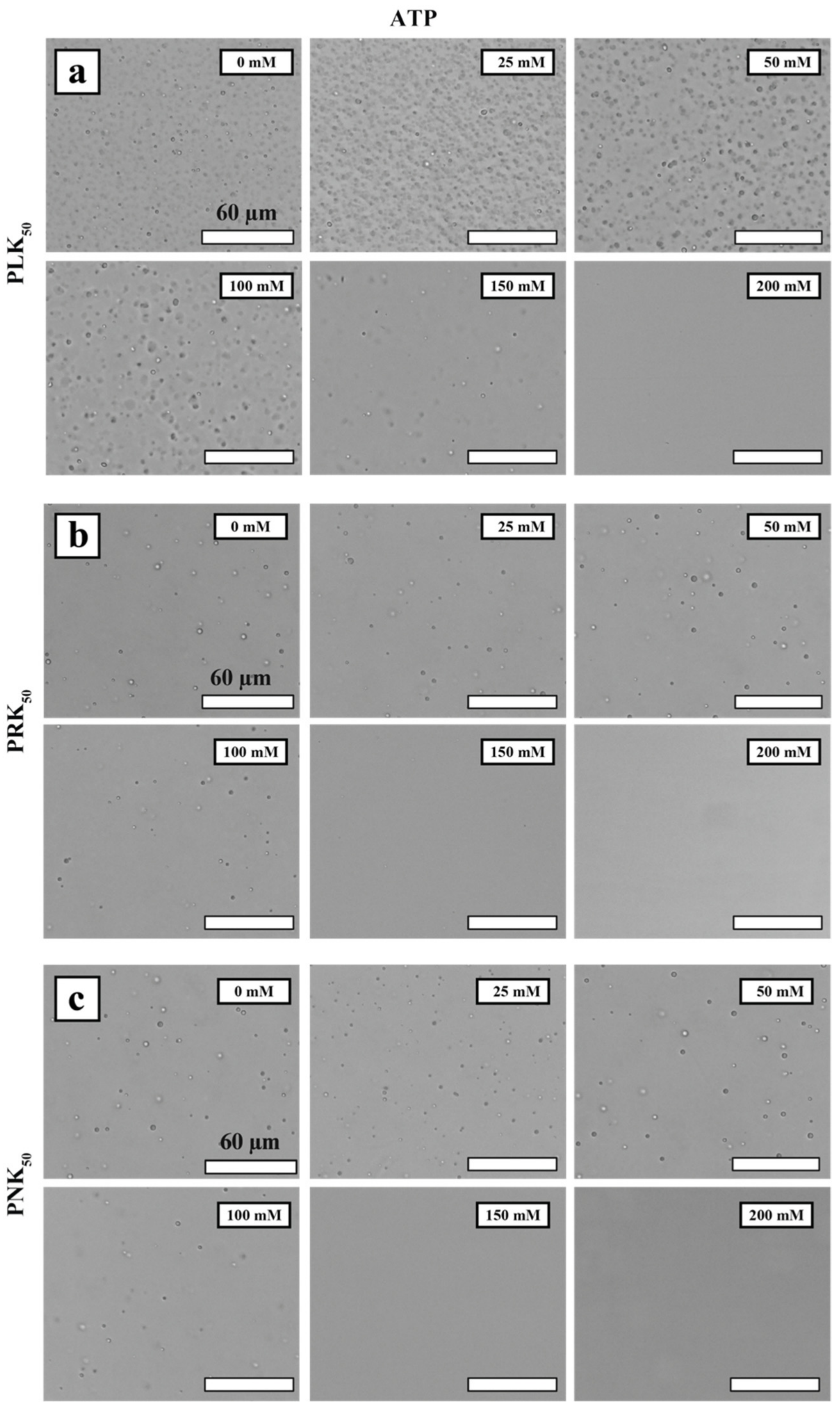

Figure S6. Micrographs of ATP with **(a)** PLK_{50}, **(b)** PRK_{50}, and **(c)** PNK_{50} complexes (charge fraction 0.5) at varying concentrations of 0, 25, 50, 100, 150, and 200 mM NaCl, highlighting morphology transitions. PLK_{50} complexes were more resistant to salt addition compared to PNK_{50} and PRK_{50} complexes. Scale bar = 60 microns in all micrographs.

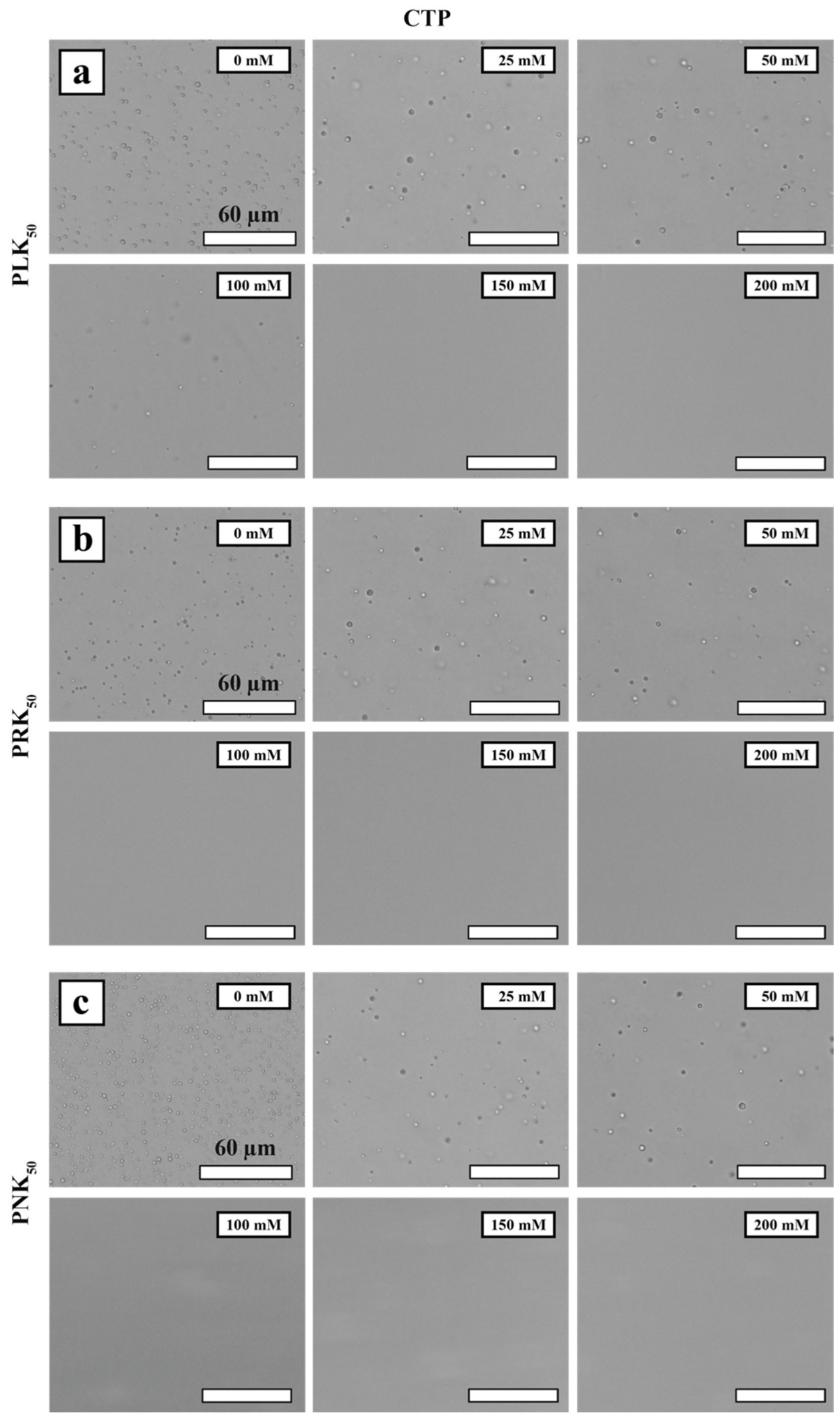

Figure S7. Micrographs of CTP with **(a)** PLK_{50}, **(b)** PRK_{50}, and **(c)** PNK_{50} complexes (charge fraction 0.5) at varying concentrations of 0, 25, 50, 100, 150, and 200 mM NaCl, highlighting morphology transitions. PLK_{50} complexes were more resistant to salt addition compared to PNK_{50} and PRK_{50} complexes. Scale bar = 60 microns in all micrographs.

References

1. Panwar, B.; Raghava, G.P.S. Prediction of Uridine Modifications in TRNA Sequences. *BMC Bioinform.* **2014**, *15*, 326. https://doi.org/10.1186/1471-2105-15-326.
2. DNA and RNA Molecular Weights and Conversions. Available online: https://www.thermofisher.com/us/en/home/references/ambion-tech-support/rna-tools-and-calculators/dna-and-rna-molecular-weights-and-conversions.html (accessed on 19 September 2025).

3. Chan, P.P.; Lowe, T.M. GtRNAdb 2.0: An Expanded Database of Transfer RNA Genes Identified in Complete and Draft Genomes. *Nucleic Acids Res.* **2016**, *44*, D184–D189.
